# The overexpression of AUF1 in colorectal cancer predicts a poor prognosis and promotes cancer progression by activating ERK and AKT pathways

**DOI:** 10.1002/cam4.3464

**Published:** 2020-10-05

**Authors:** Xin‐Yuan Tian, Jin Li, Teng‐Hui Liu, Dan‐Ni Li, Jing‐Jing Wang, He Zhang, Zhou‐Lu Deng, Fu‐Jun Chen, Jian‐Ping Cai

**Affiliations:** ^1^ Peking University Fifth School of Clinical Medicine Beijing Hospital Beijing P.R. China; ^2^ The MOH Key Laboratory of Geriatrics Beijing Hospital National Center of Gerontology Beijing P.R. China; ^3^ Graduate School of Peking Union Medical College and Chinese Academy of Medical Sciences Beijing P.R. China; ^4^ Department of General Surgery China‐Japan Friendship Hospital Beijing P.R. China; ^5^ Department of Anorectal Surgery First Affiliated Hospital of Jiamusi University Jiamusi Heilongjiang P.R. China

## Abstract

**Background:**

AUF1 is one of the AU‐rich binding proteins, which promotes rapid ARE‐mRNA degradation. Recently, it has been reported that AUF1 is involved in regulating the antioxidant system because of its capacity to bind specifically to RNA containing oxidized bases and degrade oxidized RNA. Many antioxidant proteins have been reported to be overexpressed in colorectal cancer (CRC), however, the role of AUF1 in the progression of CRC has not been explored.

**Methods:**

The expression level of AUF1 protein in human CRC cell lines and CRC tissues was detected by western blotting and immunohistochemistry (IHC. The effects of AUF1 knockdown on CRC cell proliferation, migration, invasion and changes in the signaling pathways were evaluated using a cell counting kit‐8 (CCK‐8), Transwell assays and western blotting. Subcutaneous xenograft tumor model was employed to further substantiate the role of AUF1 in CRC.

**Results:**

AUF1 protein was upregulated in CRC tissues and CRC cells, and high expression of AUF1 was significantly associated with advanced AJCC stage (*P* = .001), lymph node metastasis (*P* = .007), distant metastasis (*P* = .038) and differentiation (*P* = .009) of CRC specimens. CRC patients with the high expression of AUF1 had an extremely poor prognosis. The knockdown of AUF1 suppressed CRC cell line proliferation, migration and invasion, inhibited CRC cells tumorigenesis and growth in nude mice, and reduced phosphorylated‐ERK1/2 and phosphorylated AKT in CRC cells.

**Conclusion:**

Our findings demonstrate that AUF1 is probably involved in the progression of CRC via the activation of the ERK1/2 and AKT pathways. AU‐rich RNA‐binding factor 1 could be used as a novel prognostic biomarker and a potential therapeutic target for CRC.

## Graphical abstract

We recognized the overexpression of AU‐rich RNA‐binding factor 1 (AUF1) as a biomarker predicting a poor prognosis in colorectal cancer (CRC) patients. Overexpression of AUF1 may play a role in promoting CRC progression. AU‐rich RNA‐binding factor 1 probably promotes growth and metastasis through the ERK1/2 and AKT pathways.

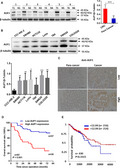

## 1 INTRODUCTION

Colorectal cancer (CRC) is one of the most frequent malignant tumors around the world.^1^ Although enormous progress in diagnosis and treatment has been made, CRC patients had bad prognosis due to the high frequency of metastasis and recurrence. Recent research has focused on personalized treatment and requires effective biomarkers for an early diagnosis and treatment. Thus, new biomarkers are greatly needed to elucidate the possible molecular mechanisms of CRC progression and as therapeutic targets.

AU‐rich RNA‐binding factor 1 (AUF1), also known as heterogeneous nuclear ribonucleoprotein D (hnRNPD), comprises a family of proteins that includes four protein subtypes p37, p40, p42, and p45, formed by selective splicing of the same mRNA precursor. AU‐rich RNA‐binding factor 1 was originally identified to accelerate degradation of polysome‐associated c‐myc mRNA.^2^, ^3^ It was reported that AUF1 regulated the expression of many key players in cancer, principally by directing the decay kinetics of their encoded mRNAs to regulate their stability,^4^ thus regulating the cell cycle, proliferation, aging and apoptosis as well as stress.^5^, ^6^ Moreover, Sekiguchi found that AUF1 can bind specifically to RNA‐containing 8‐oxo‐7, 8‐dihydroguanine, and have a role in the eliminating oxidized RNA and maintaining the gene expression related to oxidative stress states.^7^, ^8^

8‐Oxo‐7, 8‐dihydroguanine (8‐oxoG) is produced by the oxidation of guanine, and can pair with adenine and cytosine with almost equal efficiency.^9^, ^10^ The *Escherichia coli* MutT protein and its mammalian homologs, such as MTH1, MTH2, MTH3 and NUDT5, can hydrolyze a wide range of 8‐oxoG‐containing nucleotides to their monophosphates, thereby preventing the incorporation of 8‐oxoG into DNA and RNA.^11^, ^12^, ^13^ When 8‐oxoGTP is misincorporated into RNA, it may cause direct errors in translation of protein synthesis and cells may degrade the oxidized RNA with specific binding proteins, such as polynucleotide phosphorylase,^14^ YB‐1 ^15^ and AUF1. We have already found that MTH1 and NUDT5 play significant roles in the development and prognosis of CRC.^11^ Various studies have reported that AUF1 overexpression affects tumor progression in several types of cancers, including esophageal cancer, oral cancer, thyroid cancer, and lung cancer.^16^, ^17^, ^18^, ^19^ However, the role of AUF1 in the progression of CRC has not been analyzed in detail.

In this study, we investigated the difference in the expression of AUF1 in CRC tissues and CRC cell lines in comparison to adjacent normal tissues and cell lines. We found that AUF1 knockdown inhibited the proliferation and motility of CRC cells. A rescue experiment performed to overexpress AUF1 reversed these changes. Thus, we propose that AUF1 is a novel potential prognostic biomarker that is related to the progression of CRC and that AUF1 may be a new therapeutic target.

## 2 MATERIALS AND METHODS

### 2.1 Patients and specimens

Twenty pairs of CRC tissues and normal colonic tissues adjacent to carcinoma were supplied by the First Affiliated Hospital of Jiamusi University, Heilongjiang, China, from January 2014 to December 2015. All fresh surgical samples were put in the liquid nitrogen for 20 minutes and then stored at −150°C. This research was approved by the ethics committee of the First Affiliated Hospital of Jiamusi University.

The CRC tissue microarray (TMA) was brought from Shanghai Outdo Biotech Co., Ltd, which included 87 paired CRC specimens. The clinical and follow‐up information was also provided.

### 2.2 Cell lines and cell culture

CCC‐HIE‐2, HCT116, LoVo and SW620 were purchased from the China Infrastructure of Cell Line Resource. SW480, T84 and COLO320 were obtained from the American Type Culture Collection. CCC‐HIE‐2 was cultured in dulbecco's modified eagle medium (DMEM) with 20% foetal bovine serum (FBS) and 2 ng/mL EGF. HCT116, LoVo and other CRC cell lines were respectively cultured in Iscove's Modified Dulbecco's Medium, F12K and DMEM supplemented with 10% FBS.

### 2.3 Western blotting

The protein extraction and western blotting experiments were performed according to standard protocols. Antibodies used in this study were as follows: Antibody against AUF1 was purchased from Abcam. Anti‐E‐cadherin and anti‐N‐cadherin were from BD Biosciences. Anti‐Vimentin, MEK, ERK, PDK1, AKT, p‐MEK, p‐ERK, p‐PDK1, p‐AKT, and ZEB1 were from CST. Anti‐Tubulin and GAPDH antibodies were from ZSGB‐BIO. Western blotting was repeated three times.

### 2.4 Immunohistochemistry

Immunohistochemistry (IHC) staining was conducted as previously described.^11^ The TMAs were incubated with the antibody of AUF1 (1:1000 dilution). The staining results were observed using a Nikon Eclipse 80i microscope (Nikon) and the images were photographed using NIS‐Elements software.

The AUF1 immunoreactivity of 87 CRC tissues in microarray was divided into low and high expression based on the proportion and intensity staining. The proportion of positive staining cells was scored as 4 (>75%), 3 (51%‐75%), 2 (25%‐50%) and 1 (<25%). The intensity was scored as 3 (strong), 2 (moderate) and 1 (weak). The final score was calculated by multiplying the score of proportion and intensity, with 0‐6 and 7‐12 considered to represent low and high expression levels, respectively.

### 2.5 Cell transfection

To knockdown AUF1, the shRNA target sequence of the AUF1 gene was 5ʹ‐GATCCTATCACAGGGCGAT‐3ʹ. Recombinant lentiviruses expressing AUF1 shRNA or negative control shRNA (shAUF1 or shCtrl) and a rescue experiment to overexpress AUF1 again in shAUF1‐treated cells were produced by Hanbio Biotechnology. The effect of AUF1 knockdown in the transfected cells was validated by western blotting.

### 2.6 Cell proliferation by CCK‐8 assays

1 × 10^4^ transfected HCT116 and LoVo cells were seeded into 96‐well plates. After incubation for 6, 24, 48, 72 and 96 hours, 100 μL medium containing 10% cell counting kit‐8 (CCK‐8) reagent (DOJINDO) was added and incubated for 2 hours at 37°C. The absorbance of each sample was measured at 450 nm using Thermo Scientific Multiskan FC. The average of six repeated experiments was calculated.

### 2.7 Transwell assays

Complete medium containing 20% FBS was added to the bottom chambers, 2 × 10^5^ cells for silencing of AUF1 assays and 4 × 10^5^ cells for rescue of AUF1 assays in 100 μL medium without FBS were seeded into the top chambers coated with or without diluted Matrigel. After 48 hours, the cells were fixed in 4% paraformaldehyde for 30 minutes and then stained with 0.1% crystal violet solution (Beijing Solarbio Science & Technology Co, Ltd) for 1 hour. The cells that did not pass through the transmembrane containing 8 μm pores were wiped off with cotton swabs.

### 2.8 Animal experiments

For the xenograft tumor model, 6‐week‐old male BALB/c nude mice were purchased from AniKeeper and randomly divided into two groups (n = 7). 5 × 10^6^ HCT116‐shAUF1 and HCT116‐shCtrl cells were subcutaneously injected into two groups. The length and width of tumors were measured every 3 days with digital calipers and the volume was calculated using the formula: *V* (mm^3^) = 1/2 (width × length). After the nude mice were sacrificed, the weight of the tumors was measured. All experiments were approved by the Peking Union Medical College Pharmaceutical Research Institute, Beijing, China.

### 2.9 Statistical analyses

All data analyses were performed using the SPSS (version 19). The association between the AUF1 protein expression and clinicopathological features was analyzed by Pearson's χ^2^ test or Fisher's exact test. Kaplan‐Meier analysis and a log‐rank test was used to calculate and compare the overall survival. We also used both univariate and multivariate cox proportional hazards models to measure the effect of clinicopathological variables and the role of AUF1 on the prognosis of CRC patients. Student's *t* test was performed compare the means from two divided groups. *P* < .05 were considered to indicate statistical significance.

## 3 RESULTS

### 3.1 The expression of AUF1 was significantly upregulated in clinical CRC tissues and cell lines, and the high expression of AUF1 predicted a poor prognosis

**FIGURE 1:**
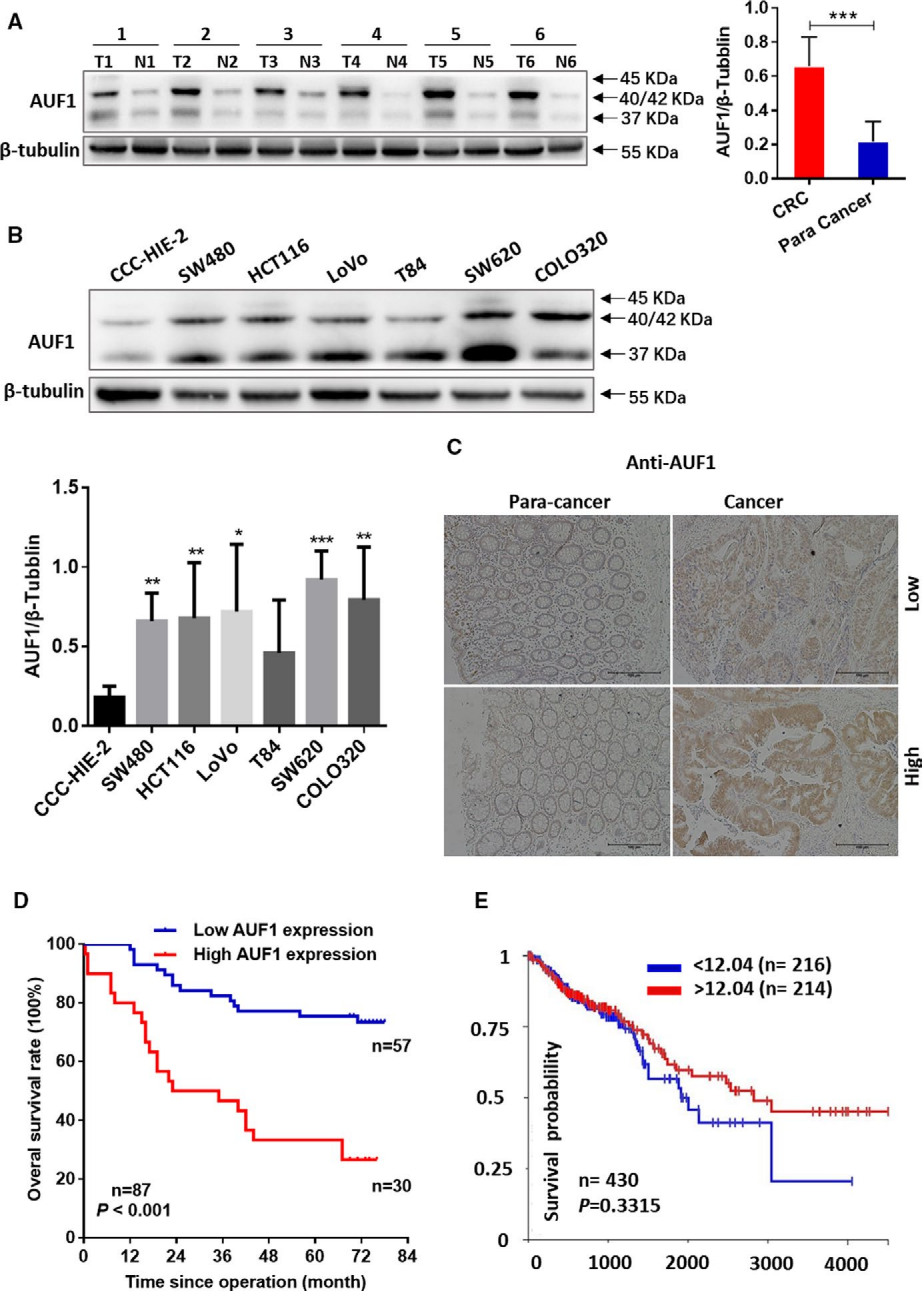
The evaluation of AUF1 in colorectal cancer (CRC) patients and CRC cell lines. A, The expression of AUF1 protein in 20 human CRC tissue (T) and paired adjacent normal tissue (N) specimens by Western blotting. The quantification of the AUF1 protein expression normalized against β‐tubulin was performed using the ImageJ software program. *P* < .0001. B, The expression of AUF1 protein in human CRC cell lines (SW480, HCT116, LoVo, T84, SW620, COLO320) and the human normal intestinal mucous cell line CCC‐HIE‐2. The quantification of the AUF1 protein expression normalized against β‐tubulin was performed using the ImageJ software program. C, The AUF1 protein expression levels in tissue microarrays, including 87 paired CRC specimens based on the intensity and proportion of immunostaining by Immunohistochemistry. D, Kaplan–Meier curves for overall survival in 87 CRC patients with high or low AUF1 expression levels (*P* < .001). E. Kaplan–Meier curves for overall survival in 430 CRC patients using TCGA database

The expression levels of the p37 and p40/p42 AUF1 isoforms in 20 paired CRC tissues were markedly upregulated compared with normal colonic tissues (Figure 1A, 0.659 ± 0.170 vs 0.218 ± 0.115, Student's *t* test, *P* < .001). Western blotting revealed two intense bands of 37, 40/42 kDa and a weakened 45 kDa band in AUF1 in these cell lines. Except for T84, the protein level of AUF1 in SW480, HCT116, LoVo, SW620 and COLO320 was significantly higher than that in CCC‐HIE‐2 (*P* < .05; Figure 1B).

To further investigate the role of AUF1 in CRC, we performed immunohistochemical staining of TMAs, including 87 paired CRC specimens. We determined differences in the AUF1 expression of tumor tissues based on the staining proportion and intensity and classified the tissue specimens into AUF1‐low and AUF1‐high groups. A relatively high AUF1 expression was observed in 30 of 87 CRC specimens, while low AUF1 expression was observed in 57 of 87. Positive immunostaining of AUF1 was relatively low in the adjacent normal tissue (Figure 1C). The results showed that the expression level of AUF1 protein in tumor tissues was upregulated compared with the paired adjacent nontumor tissues. Next, we discussed the relationships between AUF1 expression and clinical pathologic characteristics (Table 1). The expression of AUF1 was positively and significantly correlated with the AJCC stage (*P* = .001), N stage (*P* = .007), M stage (*P* = .038), and differentiation (*P* = .009). The Kaplan‐Meier analysis revealed that CRC patients with a high AUF1 expression level had significantly poorer OS than those with a low expression level (Figure 1D; *P* < .001). As shown in Table 2, a univariate analysis with a Cox proportional hazards model revealed that the following characteristics were significantly associated with poorer OS: AJCC stage (*P* < .001), N stage (*P* = .001), M stage (*P* = .006), histological differentiation (*P* = .003), and the expression of AUF1 (*P* < .001). Furthermore, a multivariate analysis indicated that the high expression of AUF1 was a significant prognostic factor for the OS rate of CRC patients (hazard ratio, 3.354; 95% confidence interval, 1.695‐6.637; *P* = .001).

**TABLE 1:** Correlation between AUF1 and colorectal cancer clinicopathological characteristics

	Total	AUF1		*P*
		low	high	
Age (y)				
<65	37	24	13	.912
≥65	50	33	17	
Sex				
Male	45	28	17	.503^a^
Female	42	29	13	
Location				
Right	42	27	15	.815^a^
Others	45	30	15	
Tumor size(cm)				
<5	37	25	12	.729^a^
≥5	50	32	18	
AJCC stage				
Ⅰ+Ⅱ	55	43	12	.001^a^, ^c^
Ⅲ+Ⅳ	32	14	18	
T stage				
T1 + T2	12	10	2	.205^b^
T3 + T4	75	47	28	
N stage				
N0	57	43	14	.007^a^, ^c^
N1 + N2	30	14	16	
M stage				
M0	84	57	27	.038^b^, ^c^
M1	3	0	3	
Differentiation				
Well + Moderate	74	53	21	.009^b^, ^c^
Poor	13	4	9	
Vascular invasion				
No	80	54	26	.228^b^
Yes	7	3	4	

a Chi‐squared test.

b Fisher's exact test.

c *P* < .05.

**TABLE 2:** Univariate and multivariate analysis of the overall survival

	Univariate analysis		Multivariate analysis	
	HR (95%CI)	*P*	HR (95%CI)	*P*
Age (y)				
<65	1			
≥65	1.178 (0.617‐2.249)	.619		
Gender				
Male	1			
Female	0.707 (0.367‐1.364)	.301		
Location				
Right	1			
Others	1.326 (0.692‐2.544)	.395		
Tumor size (cm)				
<5	1			
≥5	2.088 (0.870‐5.008)	.899		
AJCC stage				
Ⅰ+Ⅱ	1		1	
Ⅲ+Ⅳ	3.675 (1.896‐7.126)	<.001*	1.415 (1.475‐5.570)	.003*
T stage				
T1 + T2	1			
T3 + T4	1.488 (0.527‐4.201)	.453		
N stage				
N0	1		1	
N1 + N2	3.099 (1.617‐5.940)	.001*	1.134 (0.098‐13.169)	.920
M stage				
M0	1		1	
M1	5.622 (1.632‐19.372)	.006*	1.604 (0.439‐5.870)	.475
Differentiation				
Well + Moderate	1		1	
Poor	3.142 (1.472‐6.706)	.003*	1.438 (0.623‐3.320)	.395
Vascular invasion				
No	1			
Yes	0.975 (0.299‐3.176)	.967		
AUF1				
Low	1		1	
High	4.233 (2.185‐8.198)	<.001*	3.354 (1.695‐6.637)	.001*

Abbreviation: HR, hazard ratio.

a *P* < .05.

We also evaluated the prognostic role of AUF1 mRNA using The Cancer Genome Atlas (TCGA) dataset (Figure 1E, *P* = .3315), however, the mRNA level of AUF1 cannot be regarded as a prognostic index for CRC patients. This is not consistent with our conclusion that overexpression of AUF1 protein can be taken as a biomarker predicting a poor prognosis in CRC patients. This is probably because we used the protein level of AUF1, while the TCGA dataset showed the mRNA level of AUF1. Therefore, the protein levels (rather than mRNA levels available at TCGA) are necessary to determine the prognostic value of AUF1.

### 3.2 Knockdown of AUF1 inhibited CRC cell proliferation, migration and invasion

**FIGURE 2:**
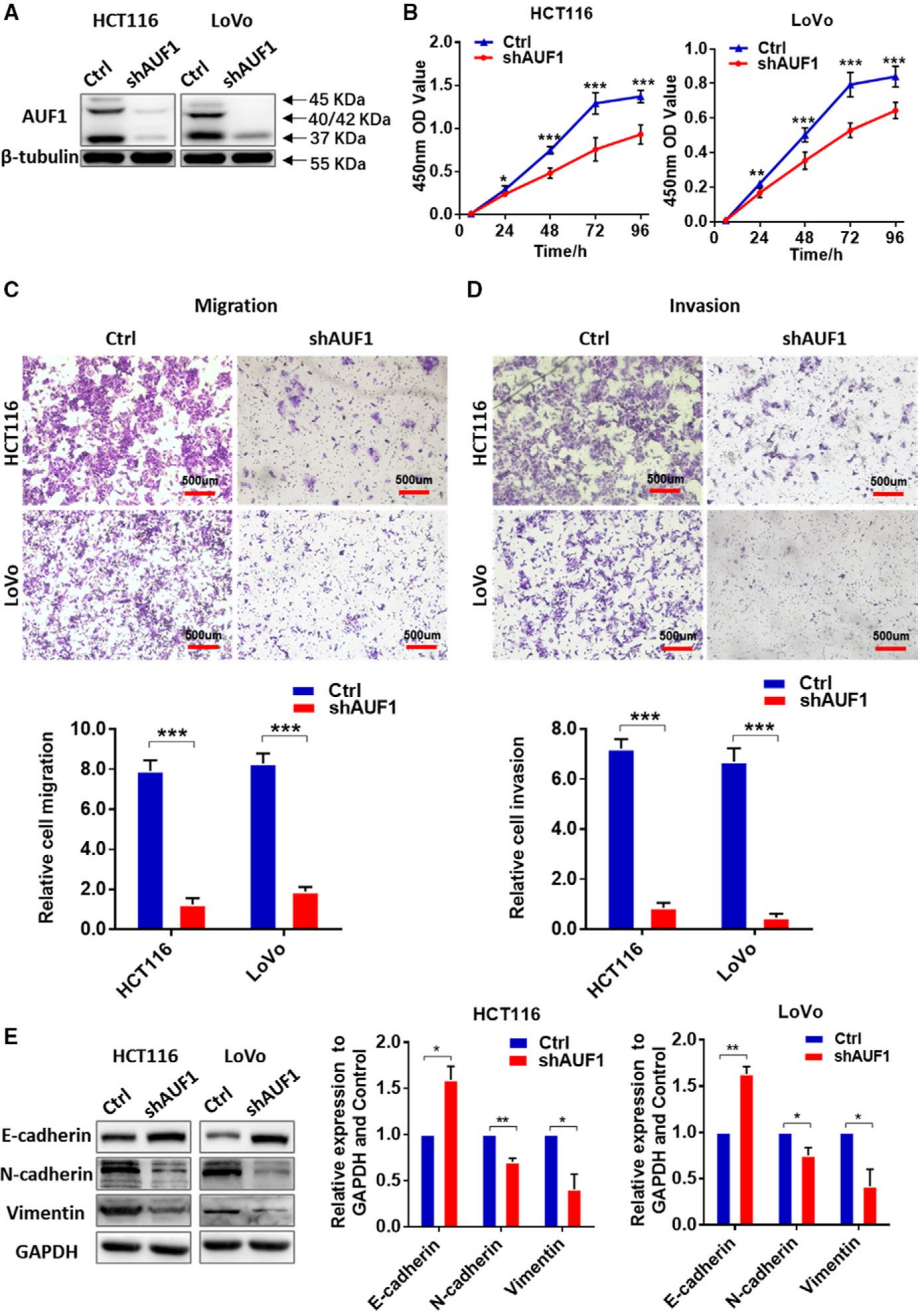
The knockdown of AUF1 inhibited the proliferation, migration and invasion of colorectal cancer (CRC) cells. A, RNAi silencing of AUF1 in HCT116 and LoVo cells. β‐tubulin was used as a control. B, Cell counting kit‐8 assays demonstrated that the knockdown of AUF1 inhibited the cell proliferation of HCT116 and LoVo cells. C and D, Transwell assays demonstrated that the knockdown of AUF1 impeded the migration and invasion of HCT116 and LoVo cells. Relative numbers of migrating and invading cells are shown. E, The knockdown of AUF1 affected the expression of epithelial‐mesenchymal transition‐related proteins

Our results showed that AUF1 was highly expressed in CRC cell lines. HCT116 and LoVo were selected to assess the biological function of AUF1 in CRC, including proliferation, migration and invasion. We knocked down AUF1 in the HCT116 and LoVo cell lines with shRNA and evaluated the knockdown efficiency by Western blotting. The shRNA targeted all isoforms of AUF1 through Blast verification and Western Blotting showed that the expression of p37, p40/42, p45 was significantly reduced (Figure 2A). The knockdown of AUF1 significantly inhibited cell proliferation in these two cell lines by CCK‐8 Assays (Figure 2B). Moreover, the cell migration and invasion assays demonstrated that the knockdown of AUF1 markedly impeded HCT116 and LoVo cell migration (Figure 2C) and invasion (Figure 2D). These results showed AUF1 deficiency can retard CRC cell progression.

### 3.3 Verification of the effect of AUF1 knockdown on CRC cells by a rescue experiment

**FIGURE 3:**
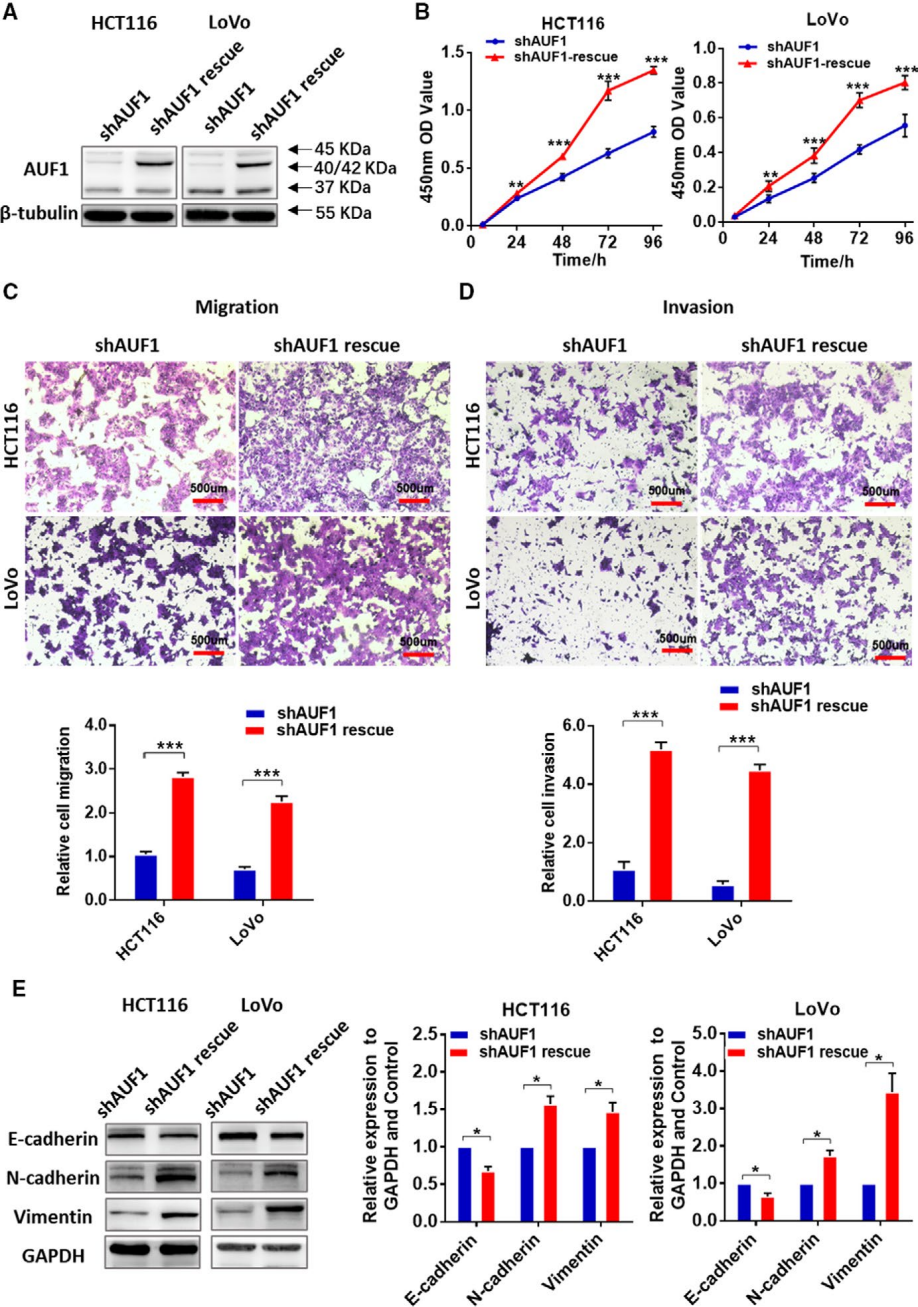
The rescue experiment used to verify the effect of the knockdown AUF1 on HCT116 and LoVo cells. A, The rescue efficiency of AUF1 in HCT116 and LoVo cells in which the expression of AUF1 had been previously knocked down. B, Cell counting kit‐8 assays demonstrated that the rescue of AUF1 repromoted the proliferation of HCT116 and LoVo cells. C and D, Transwell assays demonstrated that the rescue of AUF1 repromoted the migration and invasion of HCT116 and LoVo cells. Relative numbers of migrating and invading cells are shown. E, The rescue of AUF1 reversed the expression of EMT‐related proteins after the knockdown of AUF1

To further verify the effect of shAUF1 on CRC cells, we performed a rescue experiment using a cDNA of AUF1 that has silent mutations in the region targeted by shAUF1. The efficiency of rescue was measured by Western blotting (Figure 3A). Through the rescue experiment, the p40/42 expression level increased again in both cell lines and resulted in a reverse effect that promoted CRC cell proliferation, migration and invasion (Figure 3B‐D).

### 3.4 AUF1 affected cell motility through epithelial‐mesenchymal transition

Given that AUF1 knockdown was correlated with decreased CRC cell migration and invasion, we examined whether the epithelial‐mesenchymal transition (EMT) markers have been changed. In both AUF1‐knockdown CRC cells, Western blotting revealed the upregulation of E‐cadherin and the down‐regulation of N‐cadherin and Vimentin (Figure 2E). Opposite results were observed in HCT116 and LoVo in the rescue experiment (Figure 3E). These results revealed the effect of the expression of AUF1 on EMT, which affected the motility of CRC cells.

### 3.5 AUF1 was involved in the regulation of the ERK1/2 and AKT signaling pathways

**FIGURE 4:**
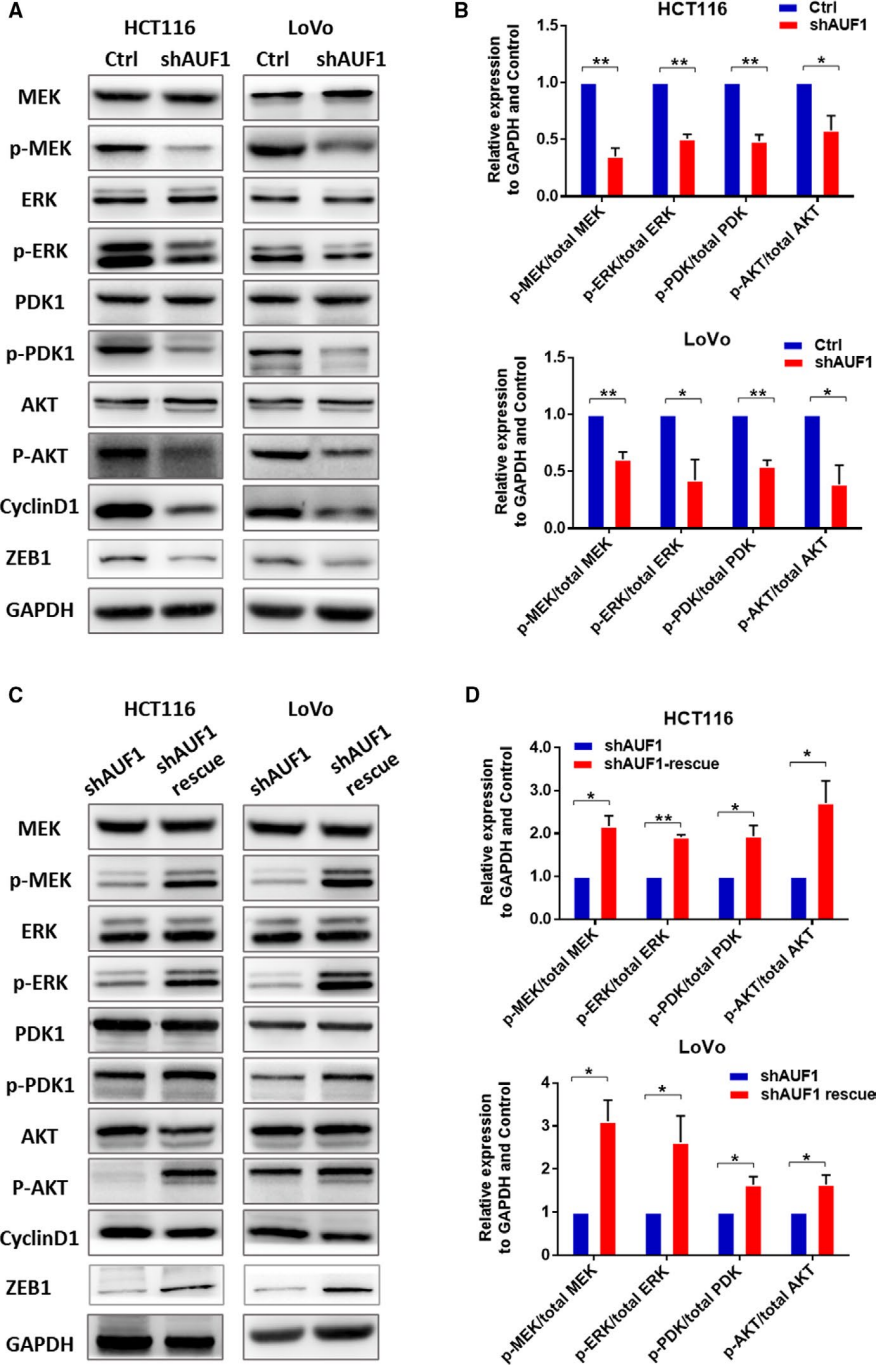
AUF1 regulated colorectal cancer (CRC) through the ERK1/2 and AKT signaling pathways. A, Western blotting to detect the expression of total MEK, phosphorylated MEK, total ERK, phosphorylated ERK, total PDK1, phosphorylated PDK1, total AKT, phosphorylated AKT and CyclinD1 in HCT116 AUF1‐knockdown cells, LoVo AUF1‐knockdown cells and control cells. B. Quantification of the expression of each protein normalized against GAPDH was performed using the Image J software program. C. Western blotting to detect the expression of total MEK, phosphorylated MEK, total ERK, phosphorylated ERK, total PDK1, phosphorylated PDK1, total AKT, phosphorylated AKT, ZEB1 and CyclinD1 in HCT116 AUF1‐rescue cells, LoVo AUF1‐rescue cells, and corresponding AUF1‐knockdown cells. D. In the rescue experiment, quantification of the expression of each protein normalized against GAPDH was performed using the Image J software program

The ERK1/2 and AKT activation was crucial for the growth and metastasis of CRC cells.^20^ Western blotting was conducted to examine the influence of AUF1 on the phosphorylation of the ERK1/2 and AKT pathways. The knockdown of AUF1 significantly reduced the phosphorylated levels of PDK1, AKT, MEK1/2, and ERK1/2 in HCT116 and LoVo cells (Figure 4A,B). Notably, the CyclinD1 and ZEB1 levels were also significantly reduced by the knockdown of AUF1. To confirm that the effects were directly dependent on the AUF1 expression, we also performed a rescue experiment, as mentioned previously. The rescue experiment showed the decreased phosphorylated levels of both MEK1/2, ERK1/2, PDK1, AKT, and ZEB1 were significantly improved by the rescue of the knockdown AUF1. However, there was no significant improvement on the level of CyclinD1 (Figure 4C,D). This approach revealed that AUF1 was associated with the activation of the ERK1/2 and AKT pathways in CRC. However, whether the knockdown of AUF1 induced the low expression of CyclinD1 remained to be discussed.

These findings suggested that the upregulation of AUF1 might affect cell proliferation, migration and invasion through the ERK1/2 and AKT pathways, which play a crucial role in CRC progression.

### 3.6 Knockdown of AUF1 limited tumorigenesis and growth in vivo

**FIGURE 5:**
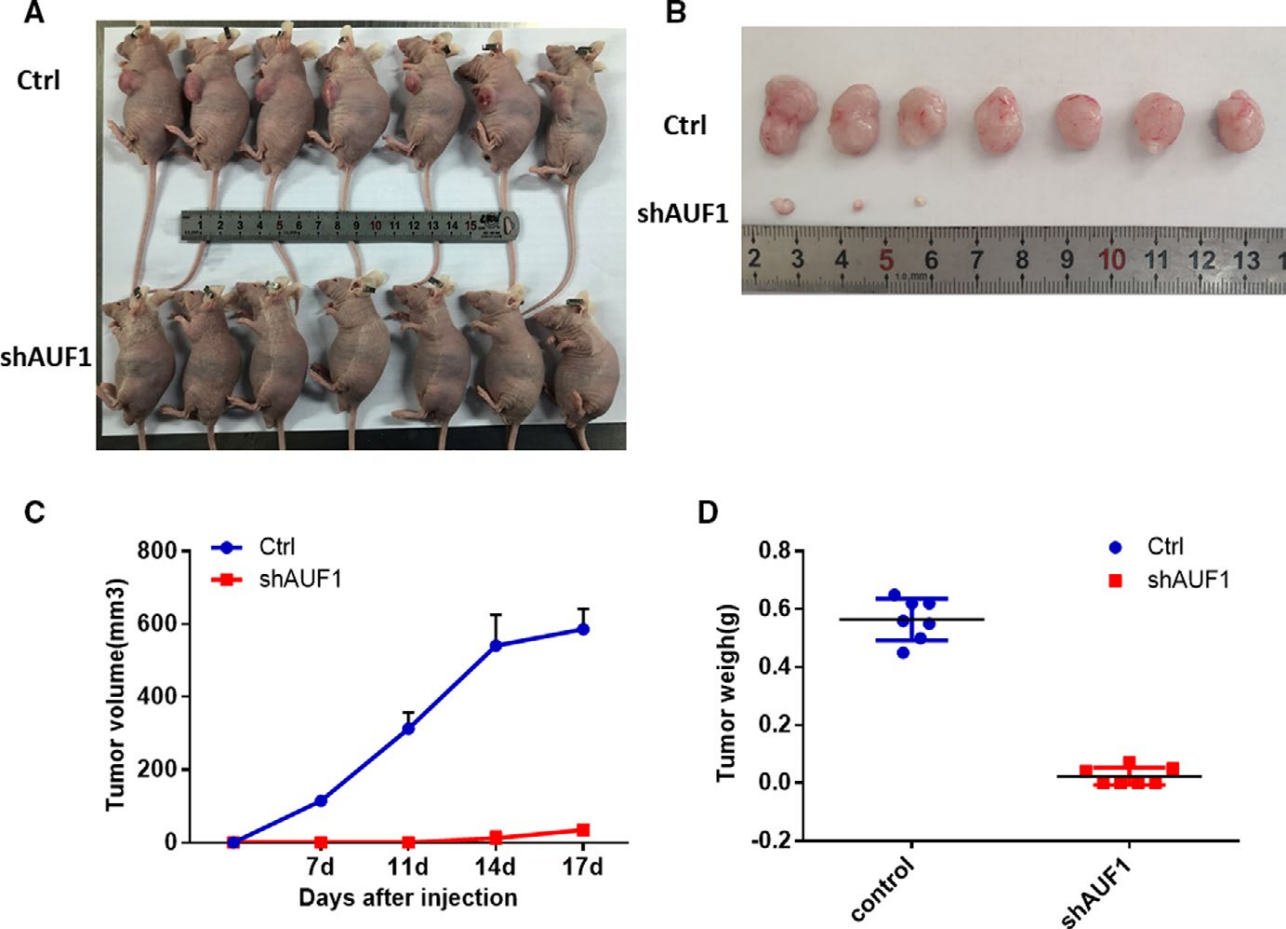
The knockdown of AUF1 induced a dramatic reduction in the growth of colorectal cancer (CRC) cells in vivo. A and B, Images of tumors formed from the subcutaneous injection of HCT116‐shCtrl and HCT116‐shAUF1 cells in nude mice (n = 7/group) and the inhibition of CRC tumorigenesis and growth by the knockdown of AUF1. C and D, The knockdown of AUF1 decreased the tumor size and weight on the 17th day

To further assess whether the knockdown of AUF1 inhibited tumor growth in vivo, we established xenograft mouse models with the subcutaneous injection of equal amounts HCT116‐shAUF1 and HCT116‐shCtrl cells in two groups (n = 7 for each group). Notably, the tumor formation in AUF1‐knockdown group was significantly reduced (3 of 7) while tumors formed in all mice in the control group (7 of 7) (Figure 5A,B). The volume and weight of the tumors that developed after the injection of HCT116‐shAUF1 cells were significantly lower in comparison to those that developed after the injection of HCT116‐shCtrl cells (Figure 5C,D). These findings were consistent with the in vitro results, demonstrating that knockdown of AUF1 inhibited CRC tumorigenesis and growth in vivo.

## 4 DISCUSSION

It is reported that AUF1 protein is overexpressed in various tumors,^16^, ^17^, ^18^, ^19^ however, the underlying mechanisms of upregulated AUF1 in CRC has not been studied in detail. We discovered that AUF1 was upregulated in CRC cell lines and tissues. Furthermore, high expression of AUF1 was significantly associated with the AJCC stage, N stage, M stage and differentiation. A Kaplan‐Meier analysis demonstrated that CRC patients with high AUF1 expression levels had poorer OS after surgical resection than those with low AUF1 expression levels and a Cox proportional hazards model indicated that AUF1 might be a potential prognostic factor in CRC.

Our results demonstrated that the upregulation of the AUF1 expression in CRC was consistent with the previously reported upregulation of AUF1 in other cancers. Several kinds of evidence support that AUF1 was involved in the initiation and development of cancer: many proteins encoded by AUF1‐targeted mRNA control pro‐ and antioncogenic processes^21^; AUF1 overexpression increased the tumor incidence in mice model^22^; AUF1 levels are enhanced in several tumors. In addition, since cancers have redox regulation dysfunction and increased ROS tension, the upregulated expression of MTH1 could be useful for preventing the incorporation of oxidized dNTP into DNA, which would therefore protect the cancer and cause progression, contributing to the poor prognosis of cancer patients.^11^, ^23^, ^24^ AUF1 is also involved in regulating the antioxidant system. As the level of oxidation increases, the expression of related proteins that protect the body from nucleic acid oxidative damage will increase and this may be one of the reasons why AUF1 is upregulated in CRC.

To clarify the biological function of AUF1 in CRC, we silenced AUF1 expression and found that the knockdown of AUF1 inhibited the proliferation, migration and invasion of HCT116 and LoVo cells. Moreover, AUF1 deficiency dramatically restrained the tumorigenesis and growth of HCT116, which were subcutaneously inoculated into nude mice. The results suggested that knockdown of AUF1 might retard the progression of CRC both in vitro and in vivo.

AUF1 consists of four related isoforms derived from a same pre‐mRNA by selective splicing of exons 2 and 7, called p37^AUF1^, p40 ^AUF1^, p42 ^AUF1^ and p45^AUF1^ according to the molecular size. Although each isoform has common RNA recognition motif (RRMs), p37 and p42 show higher affinity for AU‐rich sequences than p40 and p45.^21^ Additionally, the different functions of each isoform partly depend on the subcellular localization. When p37 and p40 shuttle from the nucleus and cytoplasm, p42 and p45 are mainly in the nucleus.^25^, ^26^ We prefer to study the AUF1 protein as a whole, therefore the shRNA we selected targeted all four isoforms and Western Blotting verified the knockdown effect.

Colorectal patients are prone to early lymph node metastasis and many patients have liver and lung metastases in the advanced stages of the disease. Epithelial‐mesenchymal transition has been recognized to be involved in tumor invasion and metastasis, including local invasion, the spread of the primary tumor, intravascular circulation, and metastasis.^27^, ^28^ The results of TMAs demonstrated that the overexpression of AUF1 was associated with lymph nodes and distant metastases. Moreover, AUF1 knockdown in HCT116 and LoVo cells caused changes in expression of EMT marker proteins. These findings suggested that the expression of AUF1 might be associated with CRC lymph node metastasis.

ERK1/2 and AKT activation was crucial for the growth and metastasis of CRC cells.^20^, ^29^ In our cell function study, the knockdown of AUF1 reduced phosphorylation of MEK1/2/ERK1/2 and PDK1/AKT in HCT116 and LoVo cells. These data indicated that upregulated AUF1 promoted the proliferation and metastasis of CRC cell lines, possibly through the activation of the ERK1/2 and AKT pathways. It was reported that the cooccurrence of alterations in the Ras/MAPK and PI3K/AKT pathways are common in CRC, which suggested that the simultaneous inhibition of the Ras/MAPK and PI3K/AKT pathways might be beneficial in the treatment of CRC.^30^ Thus, AUF1 may be a potential new therapeutic target for CRC.

Ras/MAPK and PI3K/AKT signaling pathways play an important role in cell proliferation, partly through the regulation of CyclinD1 and CDK inhibitors.^31^, ^32^ The overexpression of CyclinD1 has been described in various neoplasms.^33^ The abnormal expression of CyclinD1 can lead to cell arrest and tumor formation. It was noted that the levels of CyclinD1 decreased in two CRC cell lines with the reduced expression of AUF1. However, after the rescue experiment, the phosphorylation of the ERK1/2 and AKT pathways increased, while the expression of CyclinD1 did not. Studies have demonstrated that AUF1 regulates many cell cycle‐related genes and oncogenes, and AUF1 can destabilize CyclinD1 mRNA.^34^, ^35^ However, it was reported that although AUF1 could degrade CyclinD1 mRNA, the expression of CyclinD1 was still decreased when the AUF1 expression was knocked down^18^ and transgenic mice that overexpressed p37 AUF1 developed undifferentiated sarcomas with the concomitant overexpression of CyclinD1.^22^ These findings suggested that the regulation of the mRNA stability by AUF1 was a complex process rather than simply correlating with the level of AUF1 expression. Thus, we hypothesized that the decreased ERK1/2 and AKT pathways caused a decrease in the downstream expression of CyclinD1 after the knockdown of AUF1. After the rescue experiment, as the expression of AUF1 increased, although the ERK1/2 and AKT pathways were reactivated, CyclinD1 was not significantly upregulated in the short‐term because increased AUF1 degraded its mRNA.

Aboussekhra, A has shown that miR‐141 and miR‐146b‐5p inhibit the prometastatic mesenchymal features through repression of AUF1. This is because AUF1 can bind to and stabilize the mRNA of PDK1 and ZEB1. On one hand, down‐regulation of AUF1 decreased the levels of both total and phospho‐PDK1 and downstream AKT phosphorylation, on the other hand, down‐regulation of AUF1 repressed the expression of EMT transcription factor ZEB1, which then caused the epithelial marker E‐cadherin to be upregulated.^36^ In this study, we also found that AUF knockdown decreased the phosphorylation of PDK1 and AKT and the expression of ZEB1. Therefore, AUF1 can affect the invasion and migration as well as the proliferation capacities of colon cancer cells though EMT and AKT signaling pathway. In conclusion, we recognized the overexpression of AUF1 as a biomarker predicting a poor prognosis in CRC patients. Upregulated AUF1 may play a role in promoting CRC progression. AU‐rich RNA‐binding factor 1 probably promotes growth and metastasis through the ERK1/2 and AKT pathways. These findings also imply the significance of oxidation in the progression of cancer and will improve our understanding of the mechanisms involved in cancer, providing a novel therapeutic target for CRC.

## CONFLICT OF INTEREST

The authors declare that they have no competing interests.

## AUTHORS' CONTRIBUTIONS

CJP designed and coordinated the study, and carried out the data interpretation. TXY performed the statistical analyses and wrote the manuscript. TXY, LJ, LDN, WJJ, and ZH performed the Western blotting, CCK‐8 and Transwell experiments. TXY and LTH performed the IHC assays and scored the stained specimens. CFJ and DZL collected tissue samples. CJP carefully read and revised the manuscript. All authors read and approved the final manuscript.

## Data Availability

The data that support the findings of this study are available from the corresponding author upon reasonable request.
